# Preparation of liposomal nanocarriers containing all-trans retinoic acid and docetaxel and their evaluation in a lung cancer cell line

**DOI:** 10.1038/s41598-025-26078-x

**Published:** 2025-11-07

**Authors:** Abolfazl Sinaei, Mahmoud Osanloo, Elham Zarenezhad, Hiva Alipanah, Ali Ghanbariasad

**Affiliations:** 1 Department of Medical Biotechnology, School of Advanced Technologies in Medicine, Fasa University of Medical Sciences, Fasa, Iran; 2 Student Research Committee, Fasa University of Medical Sciences, Fasa, Iran; 3 Department of Medical Nanotechnology, School of Advanced Technologies in Medicine, Fasa University of Medical Sciences, Fasa, Iran; 4 Noncommunicable Diseases Research Center, Fasa University of Medical Sciences, Fasa, Iran; 5 Department of Physiology, School of Medicine, Fasa University of Medical Sciences, Fasa, Iran

**Keywords:** Liposome, Drug delivery, Lung cancer, Docetaxel (DTX), All-trans retinoic acid (ATRA), Biological techniques, Biotechnology, Cancer, Nanoscience and technology

## Abstract

Lung cancer remains a major global health challenge, in part because the efficacy of current treatment modalities is reduced by drug resistance and substantial adverse effects. This study assessed the suitability of liposomes as a drug delivery system to enhance the therapeutic efficacy of docetaxel (DTX) and all-trans retinoic acid (ATRA) against lung cancer. DTX- and ATRA-encapsulated liposomes were synthesized and characterized, exhibiting desirable physicochemical properties such as an appropriate particle size, a negative surface charge, and high drug encapsulation efficiency. in vitro assays using A549 lung cancer cells demonstrated that these liposomes produced markedly greater cytotoxicity compared to free drugs; additionally, these two medications acted synergistically to enhance antitumor activity. Treatment with the liposomes resulted in upregulation of the pro-apoptotic gene Bax and downregulation of the anti-apoptotic gene Bcl-2, indicating activation of apoptotic pathways. Furthermore, the liposomes inhibited cancer cell migration and invasion in vitro, suggesting that they could suppress tumor metastasis. Collectively, these findings provide evidence that liposomes containing DTX and ATRA possess favorable properties for the treatment of lung cancer and may overcome existing challenges to improve patient treatment outcomes. However, comprehensive in vivo studies are required to validate the efficacy and clinical potential of these nanocarriers.

## Introduction

Cancer represents a significant global health threat, characterized by the uncontrolled proliferation of abnormal cells that can result in fatal outcomes if not adequately addressed. Although the exact causes are not fully understood, various risk factors, both modifiable (such as tobacco use and obesity) and nonmodifiable (such as genetic mutations), contribute to the disease^1^. The World Health Organization projects more than 13 million cancer-related deaths worldwide by 2030^2^. Lung cancer is among the most lethal cancer types and remains a leading cause of cancer-related mortality, accounting for approximately 1.8 million fatalities in 2020^3^. These deaths are primarily attributable to smoking and other risk factors. Common presenting symptoms include persistent cough and chest pain^4^. Standard treatments include surgery, radiation, and chemotherapy^5^.

All-trans retinoic acid (ATRA) inhibits cancer cell proliferation and promotes differentiation without inducing the severe side effects commonly associated with traditional chemotherapies, and it has been shown to enhance anticancer effects when used in combination with appropriate chemotherapy drugs. Among the numerous treatment modalities, chemotherapeutic agents such as docetaxel (DTX) play a pivotal role in managing certain cancers, including non-small cell lung cancer. DTX disrupts microtubule dynamics, effectively hindering cell division and tumor growth; however, its clinical utility is limited by significant adverse effects that can impair patients’ quality of life, as it may also harm normal cells^6^. Moreover, all-trans retinoic acid (ATRA), a vitamin A derivative, has emerged as a promising option^7,8^. ATRA inhibits cancer cell proliferation and promotes differentiation without inducing severe adverse effects, and it has been shown to potentiate anticancer efficacy when combined with appropriate chemotherapeutic agents^9,10^.

Nanotechnology-based drug-delivery platforms offer a promising solution to the limitations of conventional chemotherapy, notably severe toxicity and drug resistance. Broadly, delivery systems are classified as organic or inorganic carriers. Organic carriers include micelles, dendrimers, and liposomes. In contrast, inorganic carriers encompass ceramic nanoparticles, carbon nanotubes, and magnetic nanoparticles. Certain nanocarriers enable targeted delivery to tumor cells, thereby enhancing therapeutic efficacy while minimizing off-target effects^11,12^. Liposomes, considered promising drug-delivery systems, possess a phospholipid bilayer that permits encapsulation of both hydrophilic and hydrophobic agents. These properties improve drug solubility, stability, and targeting while reducing adverse effects. Moreover, liposomes are biocompatible, biodegradable, and minimally toxic, rendering them suitable for therapeutic applications^13,14^.

Recently, diverse liposomal platforms have been developed to improve targeted drug-delivery efficiency. Conventional liposomes are classified based on their size, with the permeability of tumor vasculature playing a crucial role in their success^15^. The enhanced permeability and retention (EPR) effect facilitates the preferential accumulation of nanocarriers in tumor tissues due to leaky vasculature and impaired lymphatic drainage, making it a cornerstone mechanism in nanoparticle-based cancer drug delivery^16^. Additionally, PEGylated liposomes significantly increase their circulation time by enhancing stability and reducing clearance^17,18^. Newer generations, including stimulus-responsive and targeted liposomes, have been engineered to achieve more precise control of drug release and to increase cellular uptake and autophagic responses^19–22^.

It has also been demonstrated that the simultaneous or combined use of two or more drugs with differing mechanisms of action can yield advantageous effects in the treatment of various cancers, including lung cancer. For example, in a study titled “Co-Delivery of erlotinib and resveratrol via nanostructured lipid carriers: A synergistically promising approach for cell proliferation prevention and ROS-mediated apoptosis activation”, the co-delivery system demonstrated a significant enhancement in therapeutic efficacy. This was evidenced by a substantial reduction in cell viability (~ 12.6%) and a notable induction of apoptosis (~ 85.5%) in A549 lung cancer cells, attributed to the synergistic effects of erlotinib and resveratrol encapsulated in nanostructured lipid carriers^23^. Additionally, previous studies have demonstrated a synergistic effect of DTX and ATRA on DU-145 prostate cancer cells. This combination effectively induces apoptosis and downregulates anti-apoptotic gene expression, thereby rendering cancer cells more susceptible to apoptotic death and cell-cycle arrest^24^. In the present study, we investigated the effects of liposomal formulations containing ATRA, DTX, and their combination on A549 lung cancer cells. Future research endeavours must incorporate rigorous in vivo experimentation to unequivocally validate the biological efficacy and clinical viability of these nanocarriers.

## Materials and methods

### Reagents

Dimethyl sulfoxide (DMSO), isopropanol, cholesterol, docetaxel (DTX), and all-trans retinoic acid (ATRA) were purchased from Sigma-Aldrich. Phosphate-buffered saline (PBS) was obtained from Merck. RPMI medium was obtained from Bioidea. Trypsin-EDTA and penicillin/streptomycin were obtained from GIBCO (UK). Fetal bovine serum (FBS) was sourced from Shelmax. The MTT reagent was purchased from Sigma-Aldrich. The Annexin V FITC/propidium iodide (PI) detection kit was obtained from Baran Biotech. DSPC and DSPE-PEG (2000) were obtained from Avanti Polar Lipids, and sterile membrane filters (0.22 μm) were procured from Whatman. The cDNA synthesis kit was purchased from Yekta Tajzih Company (Iran).

### Preparation of liposomes

PEGylated liposomal formulations were prepared using DSPC/Chol/DSPE-PEG (2000) in a molar ratio of 64:33:3. Liposomes were prepared by the thin-film hydration method followed by extrusion. Briefly, DSPC (10 mg), cholesterol (2.5 mg), and DSPE-PEG2000 (1.8 mg) were accurately weighed and dissolved in 5 mL of chloroform together with ATRA and DTX at a 1:20 (drug: lipid, w/w) ratio. The solvent was removed by rotary evaporation under reduced pressure at 37 °C to yield a thin lipid film, which was then placed under vacuum in a desiccator overnight to ensure complete removal of residual chloroform. Liposomes were formed by hydrating the dried lipid film with 5 mL of phosphate-buffered saline (PBS; pH 7.4) for 60 min at 65 °C. The liposomal suspension was sonicated in a water bath for 30 min at 65 °C. The suspension was then extruded through a track-etch membrane (0.2 μm pore size) using a mini-extruder (Nano Afarin Pajoohan, Iran) in multiple passes at 65 °C to produce large unilamellar vesicles. Formulated liposomes were sterile-filtered through 0.22 μm membranes to reduce particle size and homogenize the samples, and were then stored at 4 °C.

### Size distribution and zeta potential determination

Particle size and size distribution were measured by dynamic light scattering (DLS) using a K-ONE NANO instrument (Korea) at 25 °C. Prior to analysis, liposomal suspensions were diluted 1:40 in deionized water and transferred to disposable cuvettes. Zeta potential, as an indicator of colloidal stability, was measured using a HORIBA SZ-100 instrument (Japan) after diluting the samples 1:40 in deionized water.

### Transmission electron microscopy (TEM)

The morphology of empty liposomes, ATRA-loaded liposomes (ATRA-LPs), and DTX-loaded liposomes (DTX-LPs) was examined by transmission electron microscopy (Philips Morgagni, 208 S, 100 kV; Netherlands). Samples were negatively stained with 2% phosphotungstic acid and air-dried at room temperature.

### Fourier transform infrared (FTIR) spectroscopy

ATR-FTIR spectroscopy was performed to evaluate the incorporation of ATRA and DTX into the liposomes. Spectra were recorded over the wavenumber range 400–4000 cm⁻¹. Spectra were acquired on a Bruker Tensor II spectrometer (Bruker Co., Germany) in attenuated total reflectance (ATR) mode.

### Evaluation of encapsulation efficiency (EE)

Encapsulation efficiency (EE%) was determined by centrifugation. Two milliliters of each formulation were centrifuged at 9,100 × g for 50 min. The supernatant, containing unencapsulated drug, was carefully collected and analyzed by UV–visible spectroscopy (CECIL CE 7250). Unencapsulated DTX and ATRA concentrations were determined at their respective absorption maxima (230 nm and 340 nm) by reference to calibration curves prepared from standard solutions in chloroform. Calibration ranges were 1.25, 2.5, 5, 10, and 20 µg/mL for DTX and 1.56, 3.125, 6.25, 12.5, 25, and 50 µg/mL for ATRA. Encapsulation efficiency was calculated as follows: EE (%) = [(Total drug − Free drug) / Total drug] × 100.

### In vitro **release studies**

The in vitro release of DTX and ATRA from liposomes was evaluated by dialysis. One milliliter of each liposomal formulation was placed in a 12 kDa molecular-weight-cutoff dialysis bag and immersed in 100 mL of PBS (pH 7.4) at 37 °C with gentle agitation^25,26^. At predetermined time points (0, 12, 24, 48, 72, and 96 h), 3 mL aliquots of the release medium were withdrawn and replaced with an equal volume of fresh PBS. Released DTX and ATRA were quantified by UV–visible spectrophotometry at 230 nm and 340 nm, respectively; concentrations were determined by reference to drug-specific calibration curves. Drug-free liposomes served as controls under all experimental conditions.

The percentage of release was calculated using the following formula:

Percentage of drug release (%) = [Concentration of drug released/Concentration of total drug] × 100

### Cell culture and in vitro cytotoxicity analysis

The lung cancer cell lines (A549) were obtained from the Iranian Pasteur Institute. RPMI-1640 medium, supplemented with 1% penicillin-streptomycin and 10% FBS, was utilized for cell culture at 37 °C, 5% CO₂, and 95% humidity in a 25 cm² flask within an incubator. Cell density was evaluated 2–3 days post-initial culture using microscopy, and subculturing was conducted when the cells reached 70–80% confluency in the T25 flasks.

Cell viability was assessed by the MTT assay. A549 cells were seeded at 1 × 10⁴ cells per well in 96-well plates and incubated overnight. Cells were assigned to six experimental groups: (1) blank liposomes, (2) DTX solution (DTX-Sol), (3) ATRA solution (ATRA-Sol), (4) DTX-loaded liposomes (DTX-LPs), (5) ATRA-loaded liposomes (ATRA-LPs), and (6) untreated control (culture medium only). Cells were exposed for 48 h to the following concentrations: DTX-Sol, 6.18, 12.37, 24.75, 49.5, 99, and 198 µM; ATRA-Sol, 15.62, 31.25, 62.5, 125, 250, and 500 µM; DTX-LPs, 2.94, 5.87, 11.75, 23.5, 47, and 94 µM; and ATRA-LPs, 18.18, 36.37, 72.75, 145.5, 291, and 582 µM. Following treatment, the medium was removed and 100 µL of MTT solution was added to each well; plates were incubated at 37 °C for 3–4 h. Formazan crystals were dissolved in 100 µL DMSO, plates were shaken for 30 min, and absorbance was measured at 570 nm^27^. Cell viability was expressed as the percentage of the untreated control. IC₅₀ values for each formulation were calculated using GraphPad Prism software and were used to select doses for combination experiments.

### Analysis of the combination index

A549 cells were treated simultaneously with varying concentrations of DTX and ATRA in both free and liposomal forms to evaluate their combined effects. The combination index (CI) for the combined treatments was calculated using CompuSyn software. According to this index, CI < 1 denotes synergism, CI = 1 denotes additivity, and CI > 1 denotes antagonism between the two agents^28^.

### Apoptosis determination by flow cytometry

Apoptosis was assessed using an Annexin V–FITC Apoptosis Detection Kit (Baran Biotech, Iran) following the manufacturer’s instructions. Briefly, A549 cells were seeded in 6-well plates and incubated for 24 h at 37 °C in a humidified atmosphere containing 5% CO₂. Cells were treated with IC₅₀ concentrations of each drug, administered either as liposomal formulations or as solutions, alone and in combination, for 48 h. Treated and control cells were harvested, washed twice with PBS, centrifuged at 550 × g for 5 min, and resuspended in 100 µL binding buffer. Annexin V–FITC and propidium iodide (PI) were added and samples were incubated in the dark for 20 min at room temperature. Samples were analyzed by flow cytometry (Miltenyi Biotech, Germany), and data were processed using FlowJo v10 software.

### RNA extraction with trizol

Total RNA was isolated from treated and control cells using TRIzol reagent (Invitrogen) according to the manufacturer’s protocol. TRIzol, a monophasic solution of phenol and guanidine isothiocyanate, disrupts cells and solubilizes cellular components, thereby preserving RNA integrity during extraction^29^. After addition of chloroform and centrifugation, the aqueous phase containing RNA was collected and RNA was precipitated with isopropanol. The RNA pellet was washed with 70% ice-cold ethanol, centrifuged to remove residual contaminants, dissolved in nuclease-free water, and incubated at 55 °C for 10 min. Extracted RNA was stored at − 70 °C. RNA concentration and purity were determined spectrophotometrically with a multimode reader (BioTek Synergy HTX) by measuring the A₂₆₀/A₂₈₀ ratio. One microgram of total RNA was reverse transcribed to cDNA using a cDNA synthesis kit (Yekta Tajhiz, Tehran, Iran), and synthesized cDNA was stored at − 20 °C until further use.

### Quantitative PCR

Relative gene expression levels were quantified by quantitative real-time PCR (qRT-PCR). Gene-specific primers were designed using NCBI BLAST and were synthesized by Bioneer Corporation (Korea) (Table 1). β-Actin was used as the endogenous reference gene. PCR reactions (20 µL total volume) were prepared according to the manufacturer’s instructions. Thermal cycling comprised an initial denaturation at 95 °C for 10 min, followed by 40 cycles of 95 °C for 15 s, 55 °C for 30 s, and 72 °C for 30 s, after which a melting-curve analysis was performed. Relative expression was calculated using the 2^−ΔΔCt method^30^.

**Table 1:** Sequences of primers used in qPCR.

Gen	Forward	Reverse
B-act	5′- CCTGCTTGCTGATCCACATCT- 3′	5′- TTCCTCCTGAGCGCAAGTAC- 3′
Bax	5′- CCCGAGAGGTCTTTTTCCGAG- 3	5′- CCAGCCCATGATGGTTCTGAT − 3′
Bcl-2	5′- CGGTTCAGGTACTCAGTCATCC 3′	5′- GGTGGGGTCATGTGTGTGG − 3′

### Scratch test

In this study, the effects of docetaxel (DTX) and all-trans retinoic acid (ATRA) on the migratory behavior of A549 cells were assessed using the scratch assay, a widely employed in vitro method to model wound healing and to evaluate cell migration and proliferation. Cells were seeded into six-well plates and grown to confluence. A linear scratch was introduced at the center of the monolayer using a sterile scalpel. Following removal of cellular debris, cells were treated with either soluble or liposomal formulations of DTX and ATRA at one-half of their IC50 concentrations. The cell-free area was monitored at predetermined time points to quantify wound closure as a measure of cell migration and proliferation. These experiments provide insights into the capacity of these agents to inhibit the cellular processes underlying cell migration^31^.

### Statistical analysis

Data analysis was performed using GraphPad Prism 10 software. One-way ANOVA was employed to determine statistical significance among different groups. Quantitative data are presented as mean ± SD. Statistical significance was considered at ***p < 0.001 and ****p < 0.0001.

## Results

### Zeta potential, size, and morphology of the pegylated liposomal nanoparticles

PEGylated liposomes were prepared from a mixture of DSPC, cholesterol, and DSPE-PEG2000, which confers structural stability and prolongs circulation time^32,33^. Previous studies have shown that PEGylation prolongs circulation time and reduces aggregation, whereas cholesterol enhances bilayer packing, decreases membrane permeability, and increases stability^34^. The hydrodynamic diameter of blank liposomes was 172 nm, with a zeta potential of − 13.3 mV. After drug loading, the mean diameters of ATRA-loaded liposomes (ATRA-LPs) and DTX-loaded liposomes (DTX-LPs) were 159 nm and 154 nm, respectively (Table 2). The corresponding zeta potentials, which serve as indicators of colloidal stability, were − 30 mV and − 18.9 mV, respectively. The particle sizes of the drug-loaded liposome formulations remained below 200 nm, a size range that favors preferential accumulation in lung tumors via the enhanced permeability and retention (EPR) effect. Furthermore, the negatively charged liposome surface is expected to prolong systemic circulation and to improve colloidal stability by reducing electrostatic interactions with plasma proteins (Table 2).

**Table 2:** Characterization of different types of liposomal NPs.

No.	Formulation	Particle size	Zeta potential (mV)
1	Blank-liposomes	172 ± 9	-13.3 ± 3
2	ATRA-LPs	159 ± 7	-30 ± 4
3	DTX-LPs	154 ± 8	-18.9 ± 4

**Fig. 1:**
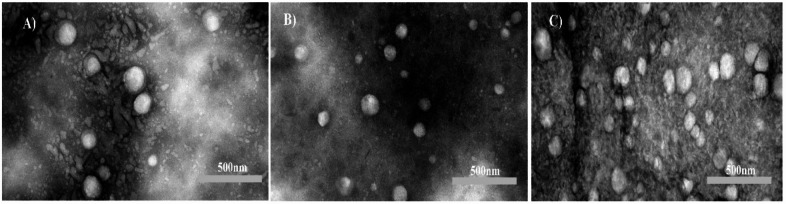
TEM image of (**A**) Blank-LPs, (**B**) ATRA-LPs, and (**C**) DTX-LPs.

We investigated the morphology of liposomes before exploring the encapsulation efficiency. The Results from TEM showed that the shapes of Blank-liposomes and drug-loaded liposomes were near spherical with uniform particle size (Fig. 1).

### FTIR spectroscopy

**Fig. 2:**
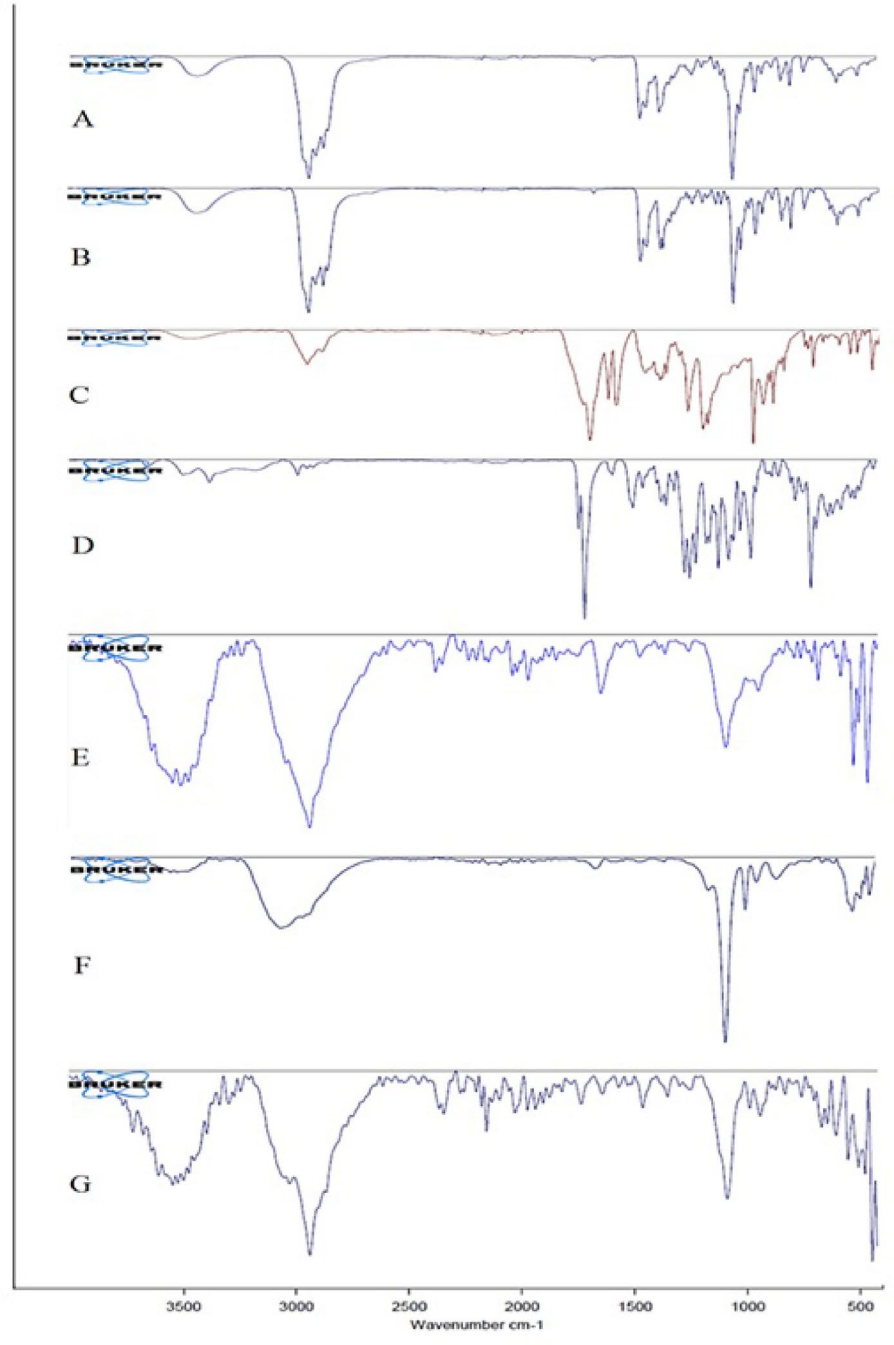
ATR-FTIR spectra of A: Cholesterol, B: DSCP, C: ATRA, D: DTX, E: Blank-LPs, F: ATRA-LPs G: DTX-LPs.

ATR–FTIR spectroscopy was employed to characterize the chemical composition and molecular structure of the samples by identifying their characteristic infrared absorption bands^35^. The ATR–FTIR spectrum of cholesterol is shown in Fig. 2A. The bands at 3427, ~ 2930, 1463, 1377, and 1053 cm⁻¹ correspond to O–H stretching, C–H stretching, CH₂ bending, CH₃ bending, and C–O stretching, respectively.

As shown in Fig. 2B, DSPC exhibits bands at 3425, ~ 2900, 1670, and 1463 cm⁻¹ that are assigned to O–H, C–H, C = O and CH₂ vibrations, respectively. The bands at 1254 and 1053 cm⁻¹ are attributed to the antisymmetric and symmetric stretching vibrations of the PO₂⁻ group, respectively, and the absorptions at 926 and 955 cm⁻¹ were assigned to the asymmetric and symmetric stretching modes of the N(CH₃)₃⁺ (choline) group in DSPC^36^.

The ATR–FTIR spectrum of ATRA is shown in Fig. 2C; peaks at 3466, 3047, ~ 2930, 1681, 1362, and 1028 cm⁻¹ were assigned to the hydroxyl group, =C–H stretching, C–H stretching, C = C, carbonyl (C = O), CH₃ bending, and C–O stretching vibration, respectively^37^.

The ATR–FTIR spectrum of DTX is presented in Fig. 2D. The band at 3489 cm⁻¹ is attributed to N–H stretching of the amide moiety, while the band at 3371 cm⁻¹ is assigned to O–H stretching. Absorptions at 1737 and 1710 cm⁻¹ correspond to carbonyl stretching vibrations, and the band at 1587 cm⁻¹ is consistent with N–H in-plane bending^38^.

The ATR–FTIR spectrum of the blank liposome (Fig. 2E) displays a broad band between 3200 and 3600 cm⁻¹ indicative of O–H stretching arising from hydrogen bonding. The band at 2925 cm⁻¹ is assigned to C–H stretching of the alkyl chains present in DSPC, DSPE-PEG2000, and cholesterol. The peak at 1741 cm⁻¹ reflects C = O stretching from ester/carbonyl groups in DSPC and DSPE-PEG2000. Phosphate and carbonyl frequencies serve as markers of head-group hydration: the band at 1247 cm⁻¹ is characteristic of PO₂⁻ stretching, and the region around 1081 cm⁻¹ is attributed to C–O–C vibrations of the PEG moiety.

The ATR–FTIR spectrum of ATRA-loaded liposomes is shown in Fig. 2F and exhibits a broad O–H band between 3248 and 3600 cm⁻¹ due to hydrogen bonding. Absorptions at 3248 and 3056 cm⁻¹ are ascribed to = C–H stretching of the alkene moiety in ATRA. Bands at 2926 and 2853 cm⁻¹ correspond to C–H stretching of alkyl chains in DSPC, DSPE-PEG2000, cholesterol, and ATRA. The band at 1658 cm⁻¹ is related to C = O stretching in ATRA, and the feature at 1585 cm⁻¹ is consistent with C = C vibrations from the phospholipid components (PC—DSPC and DSPE-PEG2000). As in the blank liposome, phosphate and carbonyl frequencies report on head-group hydration: the band at 1352 cm⁻¹ is attributed to PO₂⁻ vibrations, and the region at 1077 cm⁻¹ to C–O–C modes of the PEG moiety. Overall, ATR–FTIR spectra of ATRA-containing liposomes closely resemble those of blank liposomes, with only minor intensity differences.

The ATR–FTIR spectrum of DTX-loaded liposomes is shown in Fig. 2G; a broad O–H band is observed between 3200 and 3600 cm⁻¹, and absorptions at 3196 and 3017 cm⁻¹ are assigned to = C–H stretching associated with unsaturated moieties in DTX. The band at 2927 cm⁻¹ is attributed to C–H stretching of alkyl groups in DSPC, DSPE-PEG2000, cholesterol, and DTX. The band at 1639 cm⁻¹ corresponds to C = C stretching in DTX, while the peak at 1732 cm⁻¹ reflects C = O stretching in DTX and in phospholipid/PEG components (PC—DSPC and DSPE-PEG2000). Phosphate and carbonyl frequencies again serve as indicators of head-group hydration: the band at 1251 cm⁻¹ is attributed to PO₂⁻ stretching, and the region at 1088 cm⁻¹ to C–O–C vibrations of the PEG moiety. ATR–FTIR analyses of DTX-containing liposomes revealed spectra that are largely similar to those of blank liposomes, exhibiting only minor differences in band intensities.

### Encapsulation efficiency (EE%)

Prior to all assays, unencapsulated (free) drug molecules were removed by dialysis at 4 °C for 4 h to ensure that subsequent measurements reflected only encapsulated drug. Using the standard curve, the amount of encapsulated drug in each liposomal formulation was calculated from the measured free-drug fraction according to the equation given in Sect. 2.7. The encapsulation efficiencies were 74 ± 3% for ATRA and 52 ± 2% for DTX, indicating a higher encapsulation efficiency for ATRA in this delivery system.

### Evaluation of the release kinetics of DTX and ATRA from liposomal formulations

**Fig. 3:**
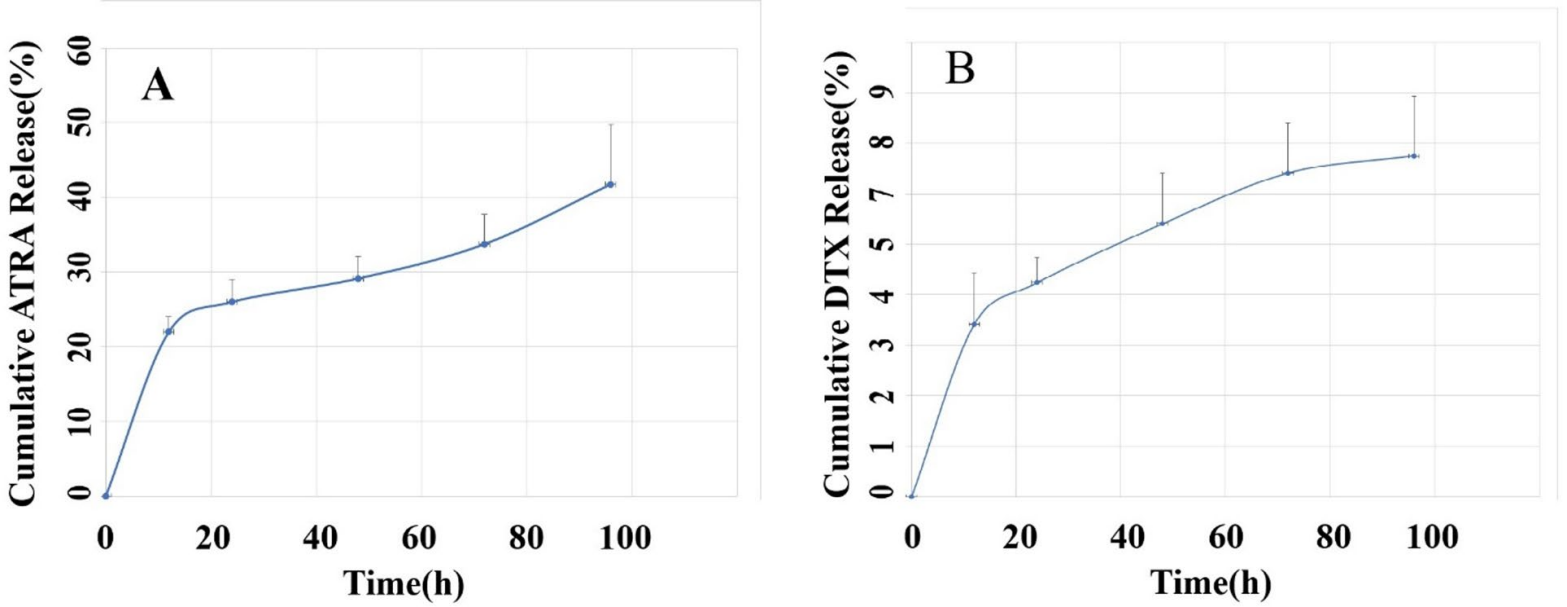
In vitro cumulative release of ATRA (**A**) and DTX (**B**) in PBS (pH 7.4) at 37 °C from drug-loaded liposomes.

Because controlled release from nanocarriers is critical to therapeutic performance, in vitro release profiles of ATRA and DTX from the liposomal formulations were determined at predetermined time points over 96 h under physiological conditions (pH 7.4 and pH 6.8, both at 37 °C). The cumulative release from each formulation at the sampled time points is depicted in Fig. 3. An initial burst release was observed for both ATRA and DTX during the first 12 h, which was followed by a sustained-release phase. By the end of the 96-h observation period, cumulative release reached 41.74% for ATRA and 6.75% for DTX. Release profiles obtained at pH 6.8 closely resembled those at pH 7.4, and no statistically significant difference was detected between the two pH conditions.

### Inhibitory effects of free and liposomal DTX and ATRA, alone and in combination, on A549 cell growth in vitro

**Fig. 4:**
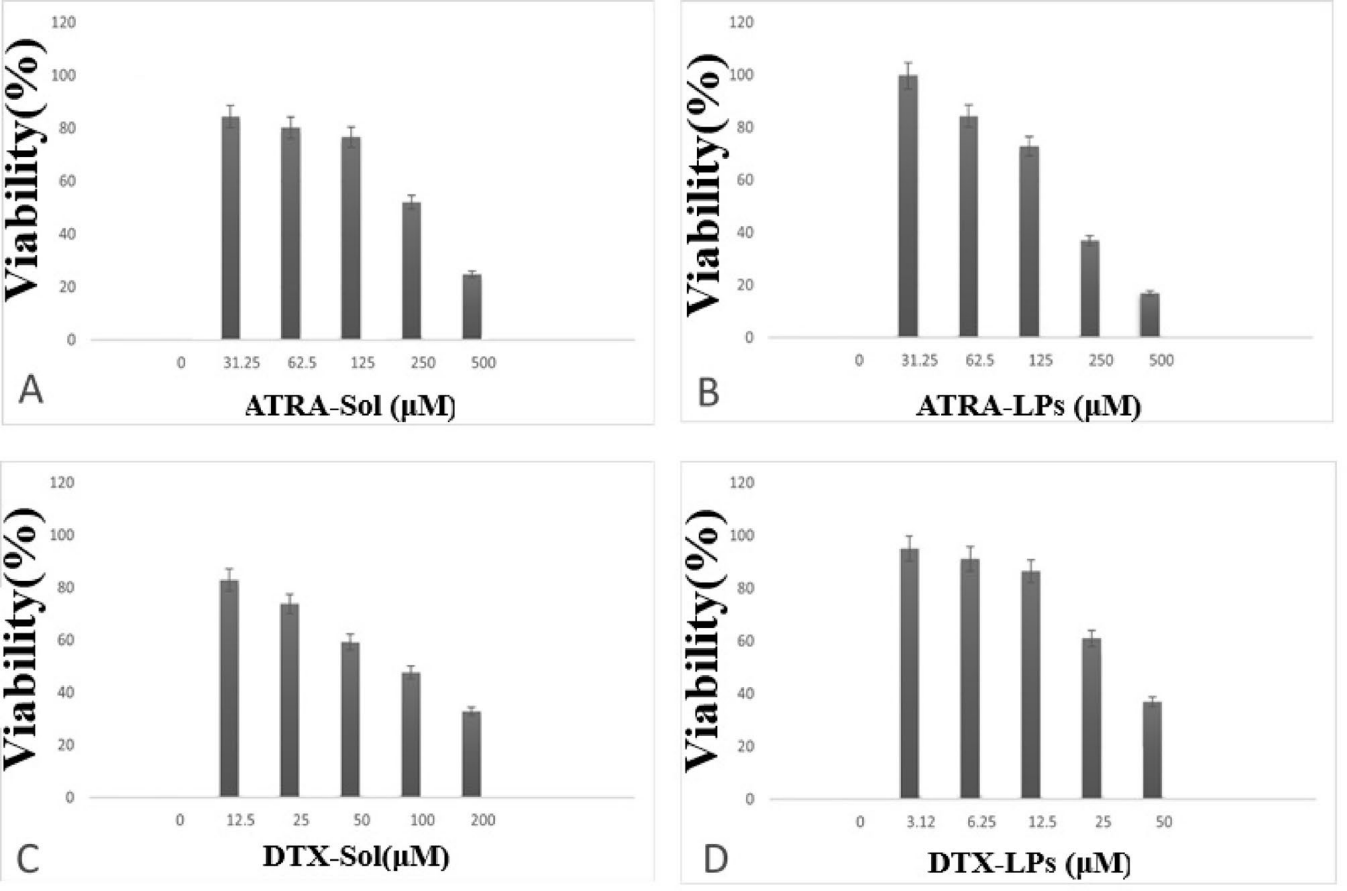
A549 cells were treated with ATRA-Sol (0–500 µM) (**A**), ATRA-LPs (0–500 µM) (**B**), DTX-Sol (0–200 µM) (**C**), and DTX-LPs (0–50 µM) (**D**) for 48 h. Cell viability was assessed by the MTT assay. All experiments were performed independently in triplicate on three separate occasions, and data are presented as mean ± SE.

**Fig. 5:**
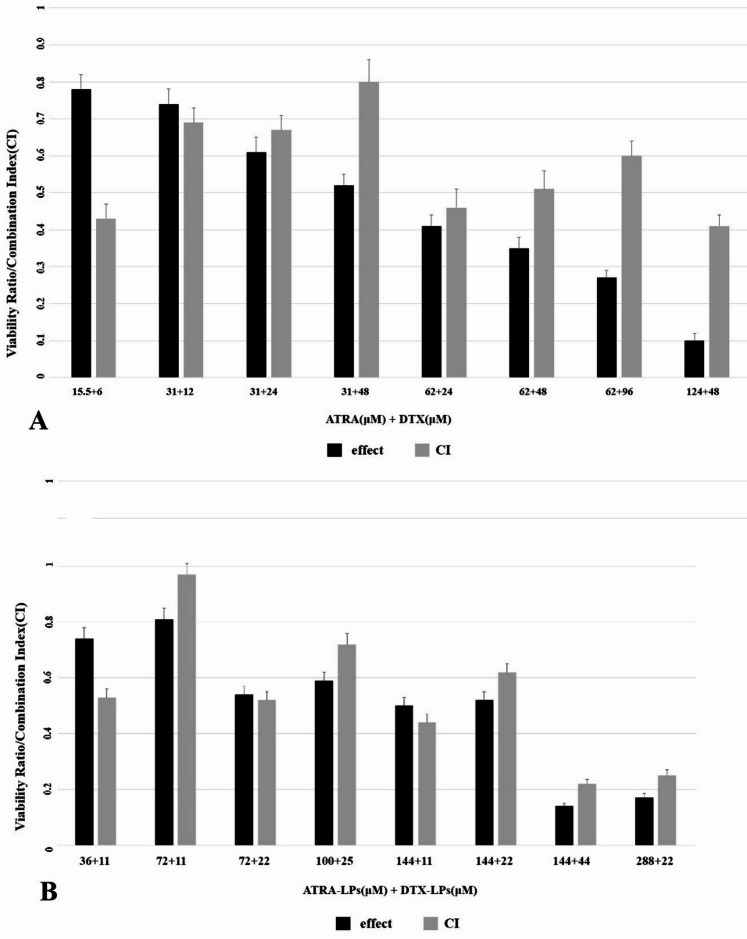
Viability ratio (effect) of A549 cells and Combination Index (CI) values for drug solutions (ATRA, DTX) (**A**) and for drug-loaded liposomes (ATRA-LPs, DTX-LPs) (**B**) in the A549 cell line.

The MTT assay was employed to evaluate the cytotoxicity of free and liposomal formulations of DTX and ATRA toward A549 cells (Fig. 4). Drug-induced effects were quantified from optical density (OD) measurements, which are inversely proportional to cell viability. IC50 values for each formulation were calculated and are presented in Table 3. For combination studies, cells were exposed to both agents concurrently, and inhibition ratios are illustrated in Fig. 5. Drug–drug interactions were quantified using the Combination Index (CI), for which a value less than one denotes synergism; that is, the combined effect exceeds the sum of the individual effects. To probe synergistic behavior, both free and liposomal combinations were tested across a range of concentration ratios.

**Table 3:** IC50 values of ATRA and DTX in solution and liposomal form.

Samples	IC50 value(µM)
ATRA – SOL	255.5 ± 3.4
ATRA – LPs	212.3 ± 1.62
DTX – SOL	81.27 ± 1.9
DTX – LPs	41.36 ± 2.3

A549 cells were treated for 48 h with varying concentrations of ATRA in combination with varying concentrations of DTX, either as free drugs or as co-administered liposomal formulations. Representative combination data are shown in Fig. 5. Overall, the liposomal formulations enhanced inhibitory effects on A549 cells across the tested concentration range. CI values below 1 at selected concentration pairs indicate synergistic interactions; notably, at lower concentrations (for example, 72 µM ATRA + 11 µM DTX), the liposomal combination produced pronounced efficacy with strong synergy, suggesting the possibility of dose reduction and concomitant decreases in toxicity.

### Analysis of gene expression levels by qRT-PCR

**Fig. 6:**
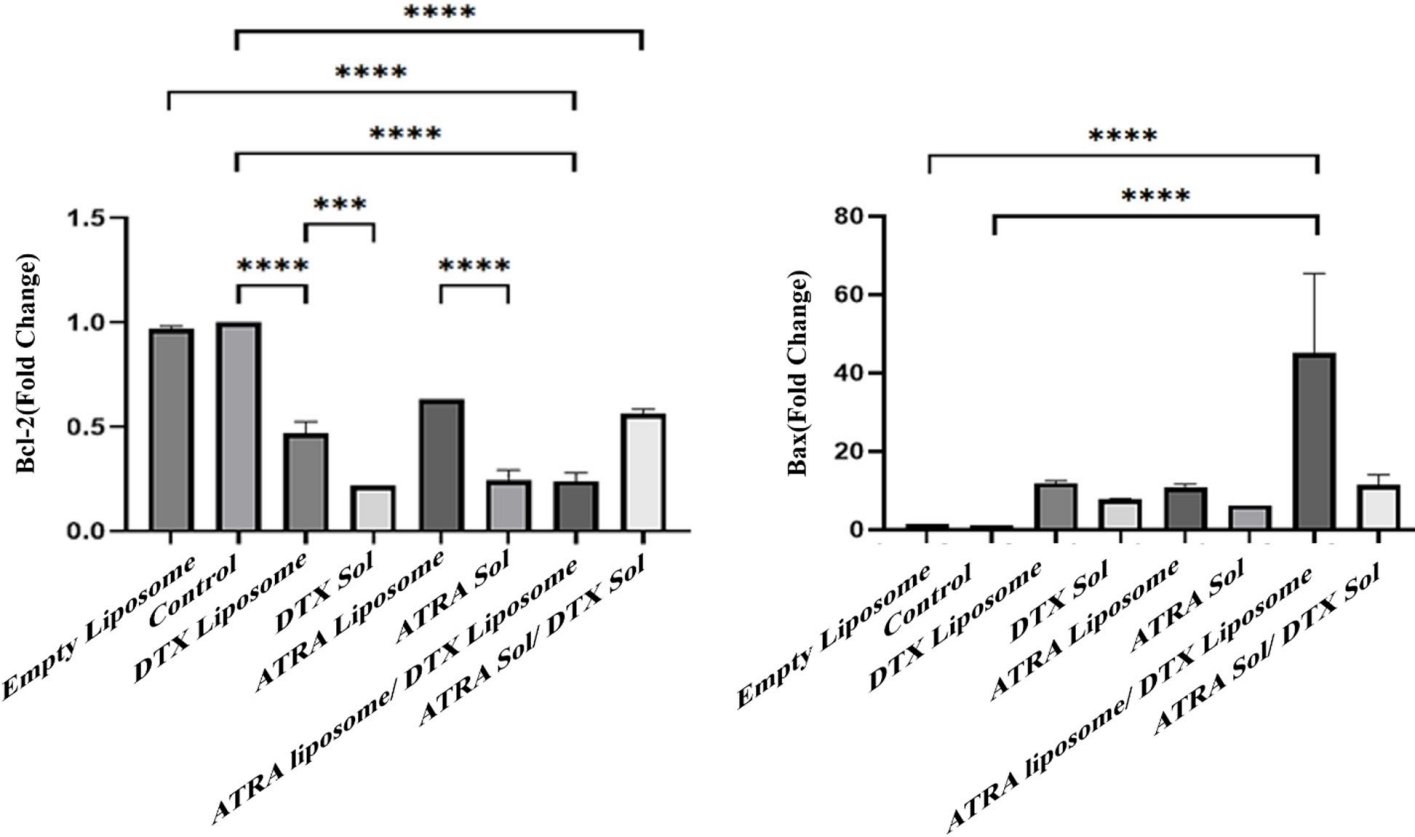
The effect of different groups on BAX and BCL-2 expression and a significant difference was shown as *P* < 0.05(***) and *P* < 0.01(****). Downregulation of the anti-apoptotic gene *BCL-2* is indicative of apoptosis and, in our study, the largest reduction was observed following treatment with the liposome mixture. Upregulation of the pro-apoptotic gene BAX indicates induction of apoptosis, with the greatest increase recorded in the liposome mixture group. Cells were treated with IC50 concentrations of the drugs in both liposomal and solution forms, as well as with their combinations, for 48 h.

The mRNA expression levels of Bax and Bcl-2, two key regulators of apoptosis, were quantified by quantitative real-time PCR. qRT-PCR was performed using gene-specific primers for Bax and Bcl-2, with β-actin serving as the endogenous reference gene. In cell groups treated with IC50 concentrations of the liposomal formulations, the solution formulations, and their combinations, Bax expression was upregulated while Bcl-2 expression was downregulated relative to untreated controls (Fig. 6). Specifically, Bax expression increased 33.88-fold in the mixed-liposome group and 11.93-fold in the DTX-LPs group compared with control; conversely, Bcl-2 expression decreased to 0.22-fold in the mixed-liposome group and to 0.24-fold in the ATRA-Sol group.

### Apoptosis was quantified using flow cytometry

**Fig. 7:**
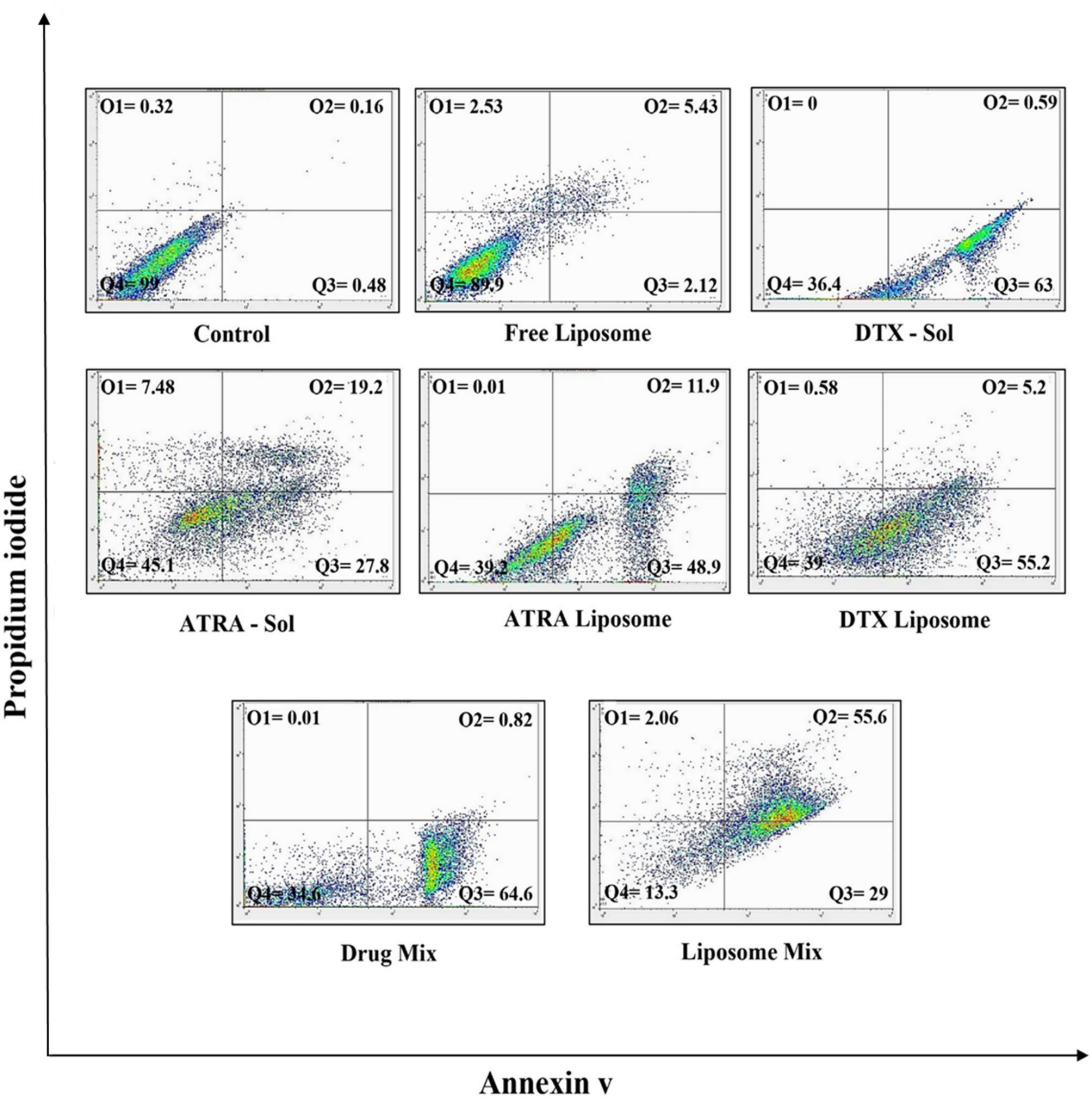
Flow cytometric analysis of apoptosis in A549 cells treated for 48 h with IC50 concentrations of the indicated formulations. Values represent the combined percentages of early and late apoptotic cells: control 0.64%; blank liposome 7.55%; DTX-Sol 63.59%; ATRA-Sol 47.00%; ATRA-LPs 60.8%; DTX-LPs 60.4%; drug mix (solution) 65.42%; and liposome mix 84.6%.

To evaluate the pro-apoptotic effects of the formulations, A549 cells were treated with IC50 concentrations of the liposomal and solution formulations and their combinations for 48 h. Apoptosis was quantified by dual staining with Annexin-V and propidium iodide (PI) followed by flow cytometric analysis. The percentages of apoptotic cells (early + late apoptosis) were: control 0.64%; blank liposome (vehicle) 7.55%; DTX-Sol 63.59%; ATRA-Sol 47.00%; ATRA-LPs 60.8%; DTX-LPs 60.4%; drug mix (solution) 65.42%; and liposome mix 84.6% (Fig. 7). These results demonstrate that all drug-containing groups induced significantly greater apoptosis than control and vehicle groups, with the liposome mix producing the most pronounced apoptotic response.

### Scratch assay results

**Fig. 8:**
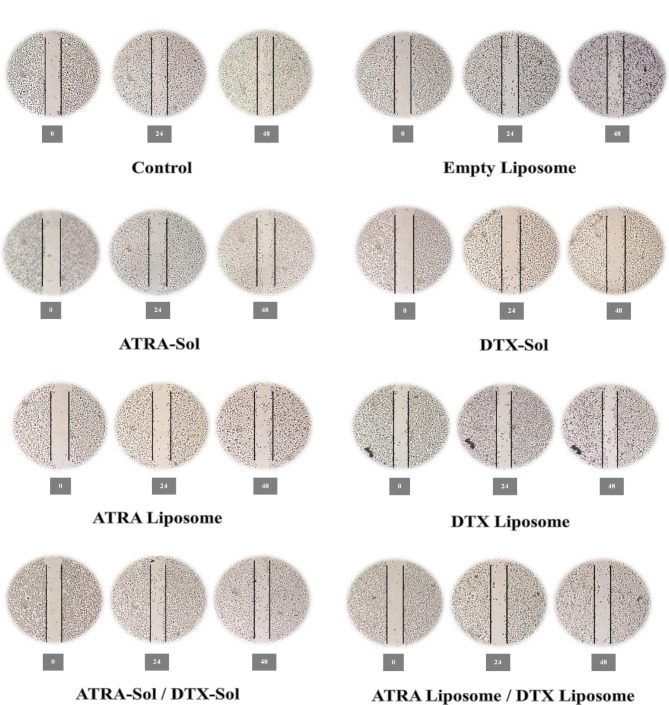
Scratch assay in A549 cells at 0, 24, and 48 h following treatment. Cells were treated with half of the IC50 concentration of each drug, administered either as free solution or as liposomal formulations, and as combined treatments; images are representative of three independent experiments.

A549 monolayers were treated with DTX and ATRA individually and in combination, and wound images were acquired at 0, 24, and 48 h to monitor wound closure. Both agents inhibited A549 cell migration and proliferation, and co-administration produced a synergistic enhancement of this inhibitory effect (Fig. 8). The liposome mix produced the most marked inhibition of wound closure. Although the scratch assay is primarily qualitative, quantitative estimation of the wound area indicated that, after 48 h, the cell-covered fraction of the initially cell-free area was approximately 20% in the DTX/ATRA-liposome group compared with 75% in the untreated control and 70% in the blank-liposome group, indicating substantially reduced migratory recovery in the liposome mix group.

## Discussion

In this study, liposomal nanocarriers encapsulating the anticancer agents ATRA and DTX were synthesized and comprehensively characterized to enhance therapeutic efficacy against lung cancer. The nanocarriers were surface-coated with polyethylene glycol (PEG) to improve their physicochemical stability and biocompatibility. Our findings demonstrated that liposomal formulations of DTX and ATRA significantly increased their cytotoxic effects on A549 lung cancer cells. Moreover, the simultaneous administration of both ATRA- and DTX-loaded liposomes produced a synergistic effect.

The nanoparticles obtained ranged from 152 to 154 nm in diameter, which lies within the optimal size range for tumor infiltration and accumulation. This result aligns with previous reports indicating that nanoparticles ≤ 200 nm can penetrate tumors via the enhanced permeability and retention (EPR) effect^39^. Furthermore, such small particle dimensions are also likely to facilitate evasion from detection and clearance by the host immune system. The observed reduction in nanoparticle size following drug loading may be attributed to factors such as alterations in the lipid architecture of the nanoparticles or specific drug–lipid interactions. The slight increase in the size of ATRA-LPs compared with DTX-LPs is presumably attributable to the inherent structural characteristics of ATRA.

The zeta potential of each liposomal formulation was measured, yielding values between − 13.3 and − 30 mV. All liposomes in this study exhibited a negative zeta potential owing to the presence of negatively charged phospholipids. Zeta potential, determined using a zeta sizer, is among the primary indicators of the stability of colloidal systems such as suspensions and emulsions. Variations in zeta potential among empty, ATRA-loaded, and DTX-loaded liposomes are mainly attributable to the distinct chemical properties of each drug. Empty liposomes derive their negative charge primarily from phospholipid phosphate groups and PEG. Loading ATRA, which contains an ionizable carboxyl group, likely increases the negative surface charge due to its acidic nature. By contrast, DTX, being more hydrophobic and neutral, alters lipid packing but does not substantially modify surface charge, thereby producing different zeta potential values. These variations arise from drug–lipid interactions and the incorporation of additional charged or neutral moieties onto the liposomal surface.

If all particles in a suspension carry a net positive or negative charge, they repel one another, thus preventing aggregation. This electrostatic repulsion is directly related to zeta potential. In general, a zeta potential exceeding ± 30 mV is indicative of excellent stability, whereas values approaching zero—representing the isoelectric point—correlate with minimal stability^40^. Based on this criterion, the liposomes developed in this study demonstrated adequate stability. Consistent with these findings, Yang et al. (2017) reported zeta potential values ranging from − 25 to − 56 mV for liposomes containing palmitoyl ascorbate and doxorubicin, either individually or in combination^41^.

ATR-FTIR spectral analysis provided detailed insights into the molecular composition and intermolecular interactions within the formulations. The presence of characteristic ATRA and DTX peaks in the respective liposomal spectra confirmed successful encapsulation. A broad OH stretching band, together with phosphate and carbonyl peaks, was observed in the liposomal spectra, indicating the formation of a stable lipid bilayer. Peaks characteristic of PEG further confirmed successful PEGylation, which is associated with enhanced stability and prolonged systemic circulation. The similarity between the spectra of the pure drugs and those of the drug-loaded liposomes suggests that the drugs were primarily incorporated within the lipid bilayer. Collectively, these results corroborate the successful development of liposomal drug delivery systems for ATRA and DTX.

Microscopic visualization remains the gold standard for defining nanoparticle morphology and structural integrity; accordingly, transmission electron microscopy (TEM) was employed in this study to examine liposomal morphology, shape, and uniformity across the synthesized formulations. TEM micrographs revealed that the liposomes—particularly the DTX-loaded formulations—were morphologically homogeneous and predominantly spherical, exhibiting a narrow and well-distributed size population.

The encapsulation efficiencies of DTX and ATRA in the liposomal formulations were 52 ± 2% and 74 ± 3%, respectively. Entrapment of a drug within liposomes depends primarily on drug polarity and solubility, together with formulation variables such as the lipid composition, buffer type and quality, drug-to-lipid ratio, and processing parameters. Hydrophobic agents, for example vinblastine, preferentially partition into the liposomal hydrophobic core and therefore typically achieve higher encapsulation efficiencies than hydrophilic drugs such as penicillin^42^; this behaviour reflects the increased affinity of hydrophobic molecules for the lipid bilayer. ML Immordino et al. reported a DTX encapsulation efficiency of ~ 58% in liposomes containing DTX and paclitaxel^43^, a value that accords with our observations. By contrast, V. M. Berlin, Grace and colleagues reported substantially higher encapsulation efficiencies (≈ 90–92%) for ATRA-LPs in a lung cancer mouse model^25^; such discrepancies in encapsulation efficiency are plausibly attributable to differences in formulation parameters and preparation methods between studies.

 in vitro release profiles were recorded over 96 h and showed that cumulative release amounted to ~ 6.75% for DTX and ~ 41.74% for ATRA from their respective liposomal formulations. ATRA, a vitamin A derivative bearing a carboxyl functional group, exhibits greater polarity than DTX, which is notably more hydrophobic; consequently, DTX tends to display more sustained release from nanoparticulate matrices under physiological conditions, a trend that has been documented in multiple reports. Our release data are broadly consistent with prior findings: for example, V. M. Berlin Grace et al. observed an ATRA release of approximately 50% from liposomes after 100 h in a mouse lung cancer model, a result comparable to ours^25^, whereas Afrouz Yousefi et al. reported a markedly slower release (~ 5%) of DTX from PEGylated liposomes after 14 days^44^. It should also be emphasized that local microenvironmental factors—such as pH, temperature, the presence of serum proteins and enzymes, and exposure to physical stimuli (e.g., ultrasound)—can substantially modulate drug release kinetics from liposomal carriers^45^.

Liposomal formulations of DTX and ATRA reduced A549 cell viability in the MTT assay (Fig. 4). As shown in Table 3, IC50 values for both liposomal ATRA and liposomal DTX were significantly lower than those of the corresponding free drugs (*p* < 0.05). Specifically, the IC50 of liposomal ATRA was reduced by approximately 43 µM (≈ 17%), whereas liposomal DTX exhibited an IC50 reduction of approximately 43 µM (≈ 49%) relative to their free counterparts. These calculated IC50 values are comparable to those reported in the literature: Bing Li et al. (2015) reported an IC50 of ≈ 0.204 mM for ATRA in A549 cells^46^, while Ke Wang et al. reported an IC50 of ≈ 17.52 µM for DTX^47^. The decrease in IC50 following liposomal encapsulation likely reflects multiple, complementary mechanisms, including increased membrane permeability, enhanced intracellular accumulation and retention, and an improved therapeutic index through reduced off-target toxicity.

Moreover, liposomal DTX (IC50 ≈ 38 µM) produced the greatest cytotoxic effect among the experimental groups (free ATRA, free DTX, and the individual liposomal forms), underscoring the potent anticancer activity of DTX and the capacity of liposomal delivery to amplify its efficacy^48^. These enhancements in potency may translate into clinically relevant advantages, such as reduced adverse effects and lower overall treatment costs. Finally, in vivo investigations have demonstrated that nanostructured lipid carriers can selectively target tumour cells while sparing normal tissues, thereby offering a favourable therapeutic index and supporting the promise of lipid-based nanocarriers for targeted cancer therapy.

Subsequent results from combined treatment with free DTX and ATRA and from the concurrent administration of ATRA- and DTX-loaded liposomes revealed that the most pronounced synergistic interaction (i.e., the lowest Combination Index) occurred in cells treated with 144 µM liposomal ATRA and 44 µM liposomal DTX. Moreover, the combined concentrations in this group were below the individual IC50 for ATRA but were within the IC50 range for DTX. These findings indicate that the liposomal co-formulation of DTX and ATRA constitutes a promising therapeutic strategy with the potential to markedly inhibit tumour growth. Consistent with prior reports, DTX and ATRA have been shown to act synergistically to induce apoptosis in DU-145 prostate cancer cells through downregulation of pro-survival genes such as survivin, MCL-1 and LTβR, which are critically involved in apoptosis regulation and cell-cycle control^24^. On the basis of these observations, subsequent experiments were directed toward the combination ratios that exhibited the strongest synergistic effects.

Flow cytometric analysis (Annexin V/PI) of A549 cells treated with IC50 concentrations of free or liposomal ATRA and DTX revealed no significant difference in apoptosis between free and liposomal DTX, whereas liposomal ATRA produced a modest but reproducible increase in apoptotic fraction relative to free ATRA. This minor discrepancy may reflect technical variability inherent to the experimental procedures. Notably, cells treated with the optimised liposomal combination exhibited the highest level of apoptosis, an outcome that is concordant with the qRT-PCR results. Collectively, the data indicate that simultaneous administration of ATRA- and DTX-loaded liposomes exerts a more potent, synergistic pro-apoptotic effect than either agent alone. These findings corroborate earlier studies: for example, R. Mhaidat et al. demonstrated that DTX induces apoptosis in melanoma cells in a concentration-dependent manner^49^, and R. Mangiarotti et al. reported that ATRA treatment progressively increases apoptotic indices over a 10-day period in a breast cancer cell line^50^.

As shown in the Results, the liposome-mix group displayed the greatest upregulation of Bax and one of the largest downregulations of Bcl-2, indicating that the combined liposomal formulation is more effective at inducing apoptosis and enhancing antitumour activity than liposomes containing each drug separately. Significant differences were observed across most comparisons, particularly between the liposome-mix group and both the control and blank-liposome groups, for expression of Bcl-2 and Bax. Subsequently, the DTX-LPs and the drug-mix (solution) groups also differed significantly from control. For Bcl-2, significant differences between liposomal and free formulations further underscore the impact of encapsulation on gene-expression responses. The absence of statistical significance for Bax in a few comparisons may be attributable to the limited sample size and number of replicates, which can reduce statistical power. This pattern is analogous to a study in which resveratrol combined with DTX produced decreased Bcl-2 expression and increased Bax expression^51^. Although Bax upregulation coupled with Bcl-2 downregulation is indicative of apoptosis, these changes alone are not definitive; therefore, to obtain more conclusive evidence of apoptotic commitment, we recommend assessing additional apoptosis-related genes and performing complementary functional assays (for example, caspase activity assays and TUNEL staining).

The results of the scratch assay demonstrated that treatment with liposomes co-containing DTX and ATRA produced a marked reduction in A549 cell migration and proliferation compared with untreated controls. This inhibitory effect exceeded that observed for the corresponding free-drug formulations. Notably, the DTX/ATRA-liposome group exhibited the most pronounced diminution in migration into the cell-free area, with wound closure reduced to approximately one-third of that measured in the control group. These observations are consistent with prior reports documenting the inhibitory effects of DTX and ATRA on cancer cell proliferation and motility^52,53^. Moreover, a 2022 scratch assay demonstrated that DTX significantly impaired cell motility in breast cancer and glioblastoma cell lines^54^. As illustrated in Fig. 8, the Liposome Mix group displayed the lowest migration rate, outperforming the Drug Mix group.

The antiproliferative and antimigratory activities of liposomes containing DTX and ATRA are likely mediated by multiple, complementary mechanisms. DTX, a microtubule-stabilizing agent, suppresses cancer cell proliferation by inducing cell-cycle arrest and activating apoptotic pathways; by contrast, ATRA promotes cellular differentiation, modulates gene expression through retinoic acid receptors, and can inhibit angiogenesis, thereby limiting tumour growth and dissemination. Collectively, these mechanisms provide a plausible mechanistic basis for the enhanced efficacy observed with the combined liposomal formulation.

We therefore recommend that future work include rigorous in vivo evaluations, the development of co-encapsulated liposomal systems for simultaneous delivery of both agents, and comprehensive assessments of therapeutic efficacy and safety in relevant animal models. To increase tumour selectivity and therapeutic index, liposomal nanoparticles may be surface-functionalized with targeting ligands (for example, peptides, antibodies, or aptamers), and dual-ligand strategies could be explored. A more detailed dissection of the combined mechanism of action of ATRA and DTX within liposomal carriers—encompassing biochemical, molecular, and cellular studies of relevant signalling pathways—would further clarify their synergistic interactions and inform rational optimisation.

This study has important limitations, the foremost being the absence of in vivo validation. It is essential to emphasize that translation of in vitro findings into clinical benefit is constrained by complex in vivo factors, including drug absorption, distribution, metabolism and excretion, as well as host immune responses, all of which can substantially influence both efficacy and safety. Consequently, comprehensive in vivo studies are required to substantiate the therapeutic potential of these nanocarriers. Moreover, although we observed upregulation of Bax and downregulation of Bcl-2 at the mRNA level following treatment, these molecular changes were not confirmed at the protein or functional level. To strengthen mechanistic conclusions, future investigations should incorporate protein-level analyses (for example, Western blotting), caspase activation assays, TUNEL staining, and detailed dose–response and time-course experiments.

## Conclusion

This study entailed the synthesis and comprehensive characterization of liposomal formulations encapsulating the anticancer agents DTX and ATRA, aimed at enhancing therapeutic efficacy in lung cancer treatment. Their combined anticancer effects were systematically evaluated against A549 lung cancer cells. The findings revealed that the engineered liposomes possessed an optimal size distribution and exhibited effective drug-loading capacities aligned with the study objectives. Crucially, liposomal formulations containing DTX and ATRA—particularly when administered simultaneously—demonstrated superior anticancer activity against A549 cells, as evidenced by significantly lower IC50 values compared to their free-drug counterparts. These formulations exerted synergistic inhibitory effects on cancer cell proliferation and migration while promoting apoptosis via multiple mechanisms, including cell cycle arrest and apoptotic pathway activation.

Taken together, the results suggest that liposomes co-encapsulating DTX and ATRA represent a promising and innovative drug delivery platform for lung cancer therapy, designed not only to enhance therapeutic outcomes but also to mitigate adverse effects, thereby potentially improving patient quality of life. This advancement constitutes a meaningful step forward in the development of lung cancer treatments.

Nevertheless, while the in vitro data underscore the cytotoxic and pro-apoptotic potential of these liposomal formulations in A549 cells, the findings remain preliminary. Extensive further research involving animal models, a broader panel of cancer cell lines, and rigorous functional assays is imperative before definitive conclusions can be drawn regarding metastasis inhibition or clinical translation.

## Data Availability

All data are contained within the manuscript, as this paper does not report data generation or analysis.
